# Epirubicin, Identified Using a Novel Luciferase Reporter Assay for Foxp3 Inhibitors, Inhibits Regulatory T Cell Activity

**DOI:** 10.1371/journal.pone.0156643

**Published:** 2016-06-10

**Authors:** Hajime Kashima, Fumiyasu Momose, Hiroshi Umehara, Nao Miyoshi, Naohisa Ogo, Daisuke Muraoka, Hiroshi Shiku, Naozumi Harada, Akira Asai

**Affiliations:** 1 Center for Drug Discovery, Graduate School of Pharmaceutical Sciences, University of Shizuoka, Shizuoka 422-8526, Japan; 2 Department of Immuno-Gene Therapy, Mie University Graduate School of Medicine, Mie 514-8507, Japan; 3 Mie University Center for Comprehensive Cancer Immunotherapy, Mie 514-8507, Japan; University of Nebraska Medical center, UNITED STATES

## Abstract

Forkhead box protein p3 (Foxp3) is crucial to the development and suppressor function of regulatory T cells (Tregs) that have a significant role in tumor-associated immune suppression. Development of small molecule inhibitors of Foxp3 function is therefore considered a promising strategy to enhance anti-tumor immunity. In this study, we developed a novel cell-based assay system in which the NF-κB luciferase reporter signal is suppressed by the co-expressed Foxp3 protein. Using this system, we screened our chemical library consisting of approximately 2,100 compounds and discovered that a cancer chemotherapeutic drug epirubicin restored the Foxp3-inhibited NF-κB activity in a concentration-dependent manner without influencing cell viability. Using immunoprecipitation assay in a Treg-like cell line Karpas-299, we found that epirubicin inhibited the interaction between Foxp3 and p65. In addition, epirubicin inhibited the suppressor function of murine Tregs and thereby improved effector T cell stimulation *in vitro*. Administration of low dose epirubicin into tumor-bearing mice modulated the function of immune cells at the tumor site and promoted their IFN-γ production without direct cytotoxicity. In summary, we identified the novel action of epirubicin as a Foxp3 inhibitor using a newly established luciferase-based cellular screen. Our work also demonstrated our screen system is useful in accelerating discovery of Foxp3 inhibitors.

## Introduction

Regulatory T cells (Tregs) play a significant role in protection against autoimmune diseases and prevention of rejection of allogenic transplants [1]. Forkhead box protein p3 (Foxp3) is a master transcription factor of Tregs and is crucial to their development and inhibitory function [2]. In humans, an autoimmune syndrome termed IPEX (immune dysregulation, polyendocrinopathy, enteropathy, X-linked) is caused by mutations in Foxp3 [3, 4]. Conversely, the immunosuppressive activity of Tregs may hamper the induction of immune responses against cancer and infectious agents. Indeed, it was shown that Tregs suppress the function of tumor-reactive T cells *in vitro* [5] and accumulation of Tregs in tumors predicts poor survival in many types of human tumors [6–9].

Many attempts have thus been made to manipulate Treg function in cancer immunotherapy and one of these approaches has involved strategies to hinder Treg-mediated immune suppressive function. Examples in the literature of compounds that act through this mechanism include the tyrosine kinase inhibitor imatinib [10], low dose cyclophosphamide [11], cytotoxic T lymphocyte antigen 4 (CTLA-4) blocking antibody ipilimumab [12] and Foxp3 inhibitory peptide P60 [13]. Among these, P60 was of particular interest due to its ability to suppress Treg function through inhibition of Foxp3 without Treg depletion [13]; a mechanism of action that is expected to have few side effects. However, compared to small molecular compounds, peptides typically do not have favorable drug-like properties when considering parameters such as stability, absorbability and cell permeability.

In this study, we established a new cell-based screen to find novel small molecular Foxp3 inhibitors. Using this system, we screened approximately 2,100 compounds and identified epirubicin, a chemotherapy drug given to treat many different types of cancer [14]. Herein, we report the mechanism of action of epirubicin as a Foxp3 inhibitor.

## Materials and Methods

### Reagents

Ten milligrams of epirubicin hydrochloride injection “NK” was purchased from Nippon Kayaku and dissolved in normal saline (Otsuka) at the time of use for *in vitro* and i*n vivo* experiments. Pirarubicin, doxorubicin, daunorubicin and idarubicin were all purchased as hydrochloride salts from Sigma-Aldrich. Recombinant human TNF-α was purchased from R&D Systems. Anti-Foxp3 and anti-GAPDH antibodies were purchased from Abcam. Anti-NFAT1 and anti-NF-κB antibodies were purchased from Cell Signaling Technologies. Anti-Foxp3 antibody for immunoprecipitation was purchased from Santa Cruz Biotechnology. Horseradish peroxidase (HRP)-conjugated anti-mouse IgG and anti-rabbit IgG antibodies were purchased from GE Healthcare. Clean-Blot^™^ IP Detection Reagent (HRP) was purchased from Thermo Scientific.

### Cell lines and culture

HEK293, a human embryonic kidney cell line (RIKEN Cell Bank) was maintained in DMEM containing 10% heat-inactivated fetal bovine serum (FBS). HEK293/NF-κB-RE cells (HEK293 stably transfected with pGL4.32 [luc2P/NF-κB-RE/Hygro] (Promega)) were maintained in RPMI containing 10% heat-inactivated FBS and 0.2 mg/mL Hygromycin B. HEK293/NF-κB-RE/Foxp3cells (HEK293/NF-κB-RE stably transfected with pcDNA3.1-Foxp3) were maintained in RPMI containing 10% heat-inactivated FBS, 0.2 mg/mL Hygromycin B and 0.5 mg/mL G418. Karpas-299, a human T cell lymphoma cell line (Public Health England) was cultured at 37°C in 5% CO_2_ in RPMI supplemented with 10% heat-inactivated FBS. CMS5a, a murine fibrosarcoma cell line from a strain of BALB/c origin [15, 16] was cultured at 37°C in 5% CO_2_ in RPMI supplemented with 10% heat-inactivated FBS and 2-mercaptoethanol.

### Reporter assays

For the NF-κB-dependent reporter assay, HEK293/NF-κB-RE/Foxp3 cells (1.5×10^4^) or HEK293/NF-κB-RE cells (1.5×10^4^) were seeded into white 96-well plates (Corning) and incubated overnight at 37°C in 5% CO_2_. These cells were treated with test drugs for 1 h. The cells were then stimulated with 0.3 ng/mL recombinant human TNF-α for 2.5 h. The medium was aspirated off and Steady-Glo (Promega) was added to the cells. The plate was then placed on a shaker for 10 min. Luminescence was detected using an ARVO Light plate reader (Perkin Elmer).

### Immunoblotting

To prepare cell extracts, cells were harvested and lysed for 30 min on ice in Phosphosafe^™^ Extra Reagent (Novagen). SDS sample buffer (4x) was added and the cell lysates were heated at 95°C for 5 min. Proteins were separated by SDS-PAGE and transferred to a PVDF membrane. After blocking in TBS (pH 7.6) containing 3% skim milk, the membrane was incubated with a primary antibody. After washing three times with TBS, the membrane was incubated with a secondary antibody. After washing an additional three times, signals were detected using ECL^™^ Prime Western Blotting Detection Reagent (GE Healthcare). The amount of detected proteins was determined by quantitation of the band intensities using Multi Gauge ver 3.2 (Fuji Film).

### Immunoprecipitation

Cell lysates were prepared in the same manner as for immunoblotting experiments. The lysates were incubated with anti-Foxp3 antibody at 4°C for 4 h and then mixed with Protein G Plus / Protein A Agarose Suspension (Calbiochem). After overnight incubation at 4°C, the immunoprecipitates were washed four times with PBS, resuspended in SDS sample buffer and heated at 95°C for 5 min. *In vitro* immunoprecipitation (cell-free assay) was performed by incubating the lysates with epirubicin and anti-Foxp3 antibody at 4°C for 4 h.

### Mice

Seven to 8 week-old female BALB/c (H2d) mice were obtained from Japan SLC and housed under specific pathogen-free conditions at the Animal Center of Mie University. Experimental protocols were approved by the Animal Ethics Committee of Mie University, Tsu, Japan.

### T cell proliferation and suppression assays

Spleen isolated from BALB/c mice was dissociated and subjected to isolation of CD4^+^CD25^+^ or CD8^+^ T lymphocytes using magnetic cell sorting with mouse CD4^+^CD25^+^ T regulatory cell or CD8^+^T cell isolation kits and an autoMACS separator (Miltenyi Biotec). CD4^+^CD25^+^ T lymphocytes were seeded in round-bottom 96-well plates coated with anti-CD3ε antibody (1μg/mL, eBioscience). Anti-CD28 antibody (1 μg/mL, eBioscience), IL-2 (60 IU/mL, provided by Novartis) and epirubicin were then added to each well. After incubation at 37°C for 48 h, the activated CD4^+^CD25^+^ T cells were harvested. Using round-bottom 96-well plates coated with anti-CD3 antibody (1 μg/mL), CD8^+^T cells (4×10^4^) stained with carboxyfluorescein succinimidyl ester (CFSE) were cocultured with the harvested CD4^+^CD25^+^ T cells (4×10^4^) in the presence of anti-CD28 antibody (1 μg/mL) at 37°C for 72 h. The cells were harvested, stained with anti-CD8-APC antibody (eBioscience) and analyzed using a FACS Canto II flow cytometer (Becton Dickinson).

### Mouse *in vivo* assays

At day 0, female BALB/c mice (8 mice per group) were inoculated subcutaneously with CMS5a cells into the right inguinal region. Epirubicin (0.1, 0.3 or 1 mg/kg) or saline was given at days 3, 5 and 7 intravenously. On day 8, mice were euthanized and tumors were removed. Tumor-infiltrating lymphocytes (TIL) were dissociated from tumors using a gentleMACS dissociator according to the manufacturer’s instructions (Miltenyi Biotec). The collected cells were seeded in 24-well plates and stimulated with phorbol 12-myristate 13-acetate (PMA) and Ionomycin at 37°C for 1 h, and then cultured for 6 h with GolgiPlug^™^ (BD Biosciences). The cells were harvested and stained with PreCP-Cy^™^5.5 rat anti-mouse CD4 antibody (BD Pharmingen) and V500 rat anti-mouse CD8a antibody (BD Horizon) at 4°C for 15 min. The stained cells were fixed with Fixation/Permeabilization Concentrate and Diluent (1:3, eBioscience) at 4°C overnight. After washing, Permeabilization Buffer (eBioscience) was added and the fixed cells were stained with PE-conjugated anti-mouse/rat Foxp3 (eBioscience), anti-mouse IFN-γ-APC (eBioscience), and PE-conjugated anti-mouse IL-2 (BioLegend) antibodies. The stained cells were analyzed using a FACS Canto II flow cytometer (Becton Dickinson).

### Statistical analysis

Data are represented as mean ± standard deviation (SD), and were analyzed using a Student’s *t* test or Dunnett's test. P values of *p*<0.05 were considered statistically significant.

## Results

### Development of a new cell-based screen that detects the reversal of NF-κB reporter signal suppression by Foxp3

**Fig 1:**
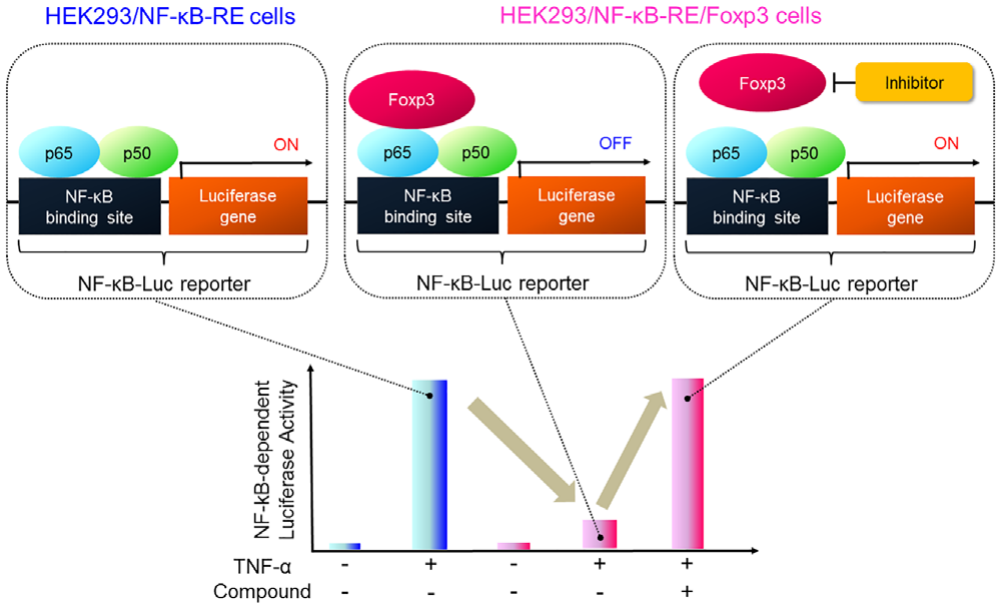
Schematic presentation of a novel reporter assay system to find Foxp3 inhibitors.

It has been reported that HEK 293 cells transiently transfected with Foxp3 have reduced basal NF-κB activation or reduced NF-κB activation in response to TNF-α stimulation [17]. Based on that finding, we designed a new cell-based screen that detects reversal of this suppression of NF-κB reporter signal by Foxp3 (Fig 1). To apply this approach to screening, we first stably co-transfected HEK 293 cells with a NF-κB-luciferase reporter plasmid and a plasmid encoding Foxp3 cDNA. As shown in S1 Fig, Foxp3 mRNA expression levels in the resultant HEK293/NF-κB-RE/Foxp3 cells was higher than that in HEK293 cells transiently transfected with Foxp3. In addition, we used peptide P60 as a reference compound to validate these cell lines as an assay system and confirmed that P60 restored Foxp3-inhibited NF-κB activity in a concentration-dependent manner, in consistent with the previously reported result [13] (S1 Fig). After the optimization of screening conditions such as the concentration of TNF-α, a stimulator of NF-κB and stimulation time (data not shown), inhibitor screenings were initiated in a 96-well plate formats.

### Epirubicin represses the inhibitory effect of Foxp3 on the activity of NF-κB

**Fig 2:**
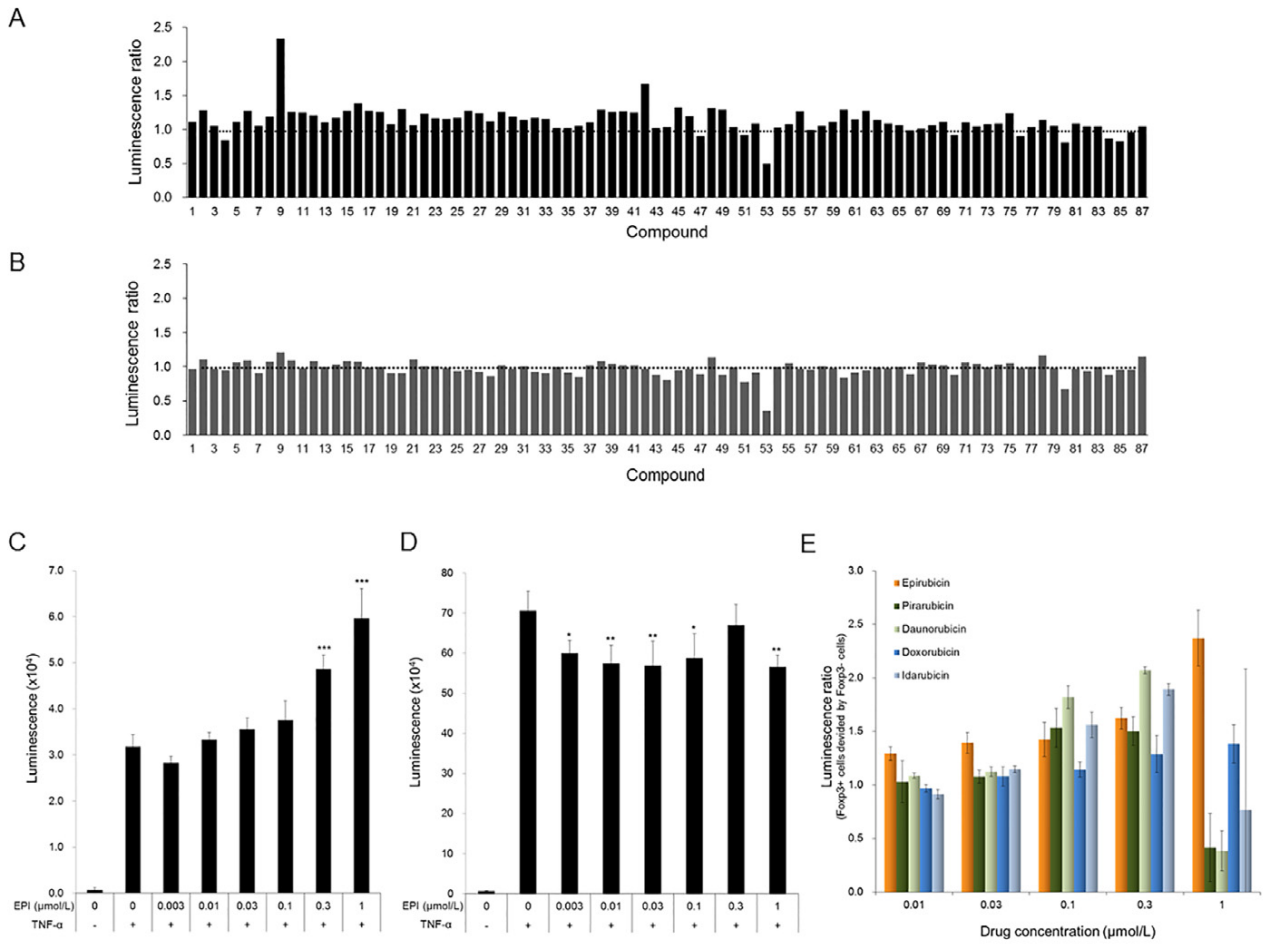
Epirubicin (EPI) represses the inhibitory effect of Foxp3 on the activity of NF-κB. (A) HEK293/NF-κB-RE/Foxp3 cells. (B) HEK293/NF-κB-RE cells. About 90 compounds were screened in duplicate. The cells were pretreated with each compound (1 μmol/L) for 1h and then stimulated with 0.3 ng/mL TNF-α for 2.5h, followed by detection of NF-κB-dependent luciferase activity. Normalized activity is expressed as the fold difference relative to the control activity (stimulated cells pretreated with DMSO). Each column represents the mean (n = 2). Compound 9 and 53 indicate epirubicin and digoxin, respectively. (C) HEK293/NF-κB-RE/Foxp3 cells. (D) HEK293/NF-κB-RE cells. The cells were pretreated for 1 h with epirubicin (EPI, 0.003–1 μmol/L) and then stimulated with 0.3 ng/mL TNF-α for 2.5 h, followed by detection of NF-κB-dependent luciferase activity. Each column represents the mean ± SD (n = 3). Asterisks represent statistically significant differences from DMSO-treated control as determined by Dunnett’s test (*, *p*< 0.05; **, *p*< 0.01; ***, *p*< 0.001). (E) HEK293/NF-κB-RE/Foxp3 or HEK293/NF-κB-RE cells were pretreated for 1 h with the indicated drug (0.01–1 μmol/L) and then stimulated with 0.3 ng/mL TNF-α for 2.5 h, followed by detection of NF-κB-dependent luciferase activity. After calculating normalized activity relative to the control (stimulated cells pretreated with DMSO) in each cell line, the luminescence ratio in HEK293/NF-κB-RE/Foxp3 cells (Foxp3+ cells) was divided by that in HEK293/NF-κB-RE cells (Foxp3- cells). Each column represents the mean ± SD (n = 3).

To evaluate the effectiveness of our luciferase-based assays in identifying useful lead inhibitors that selectively inhibit the function of Foxp3, we first screened about 90 compounds from our reference library made up of compounds with proven pharmacological activities. Data from the primary screen of all compounds at 1 μmol/L are shown as fold changes from the basal level in Fig 2A and 2B. Compound 9 selectively induced the expression of the luciferase reporter in HEK293/NFκB-RE/Foxp3 cells and was identified as a potential hit. This compound was epirubicin, a type of anthracycline, the chemical structure of which is shown in S2 Fig. Compound 53 reduced the expression of the luciferase reporter in both cells. This compound was digoxin, cardiac glycoside drug, which has inhibitory effect of NF-κB signaling [18]. This result, therefore, indicates that this assay system works well. We next performed concentration–response studies of epirubicin using this assay system, and epirubicin demonstrated concentration-dependent induction of expression of the luciferase reporter specifically in HEK293/NF-κB-RE/Foxp3 cells (Fig 2C versus 2D). Epirubicin had no influence on cell viability under the tested reporter assay conditions (data not shown). We screened an additional approximately 2,000 compounds from our small molecular chemical library but were not able to find additional compounds that recovered reporter activity more than did epirubicin (data not shown). Anthracyclines are well-known cancer chemotherapeutic agents and the spectrum of their antitumor activity is changed by substitutions of the anthraquinone segment or the carbohydrate unit [19]. We therefore investigated the structure–activity relationships of the clinically used anthracyclines doxorubicin, pirarubicin, daunorubicin and idarubicin (S2 Fig). Data was shown as the normalized reporter activity in HEK293/NF-κB-RE/Foxp3 cells divided by that in HEK293/NF-κB-RE cells (Fig 2E). Pirarubicin, daunorubicin and idarubicin indicated a tendency to increase reporter activity, as seen with epirubicin, but had weaker activity than epirubicin. At the high concentration of 1 μmol/L, pirarubicin, daunorubicin and idarubicin decreased the reporter activity. Since these anthracyclines had no influence on cell viability under these reporter assay conditions (data not shown), they might act directly on the luciferase reporter gene at such a high concentration. The structural difference between epirubicin and the other 4 anthracyclines is the steric position of the amino sugar 4'-OH. Our data may therefore suggest that this moiety is required to restore Foxp3-inhibited NF-κB activity. We thus chose to conduct further characterization of epirubicin.

### Epirubicin blocks the physical interaction between Foxp3 and p65

**Fig 3:**
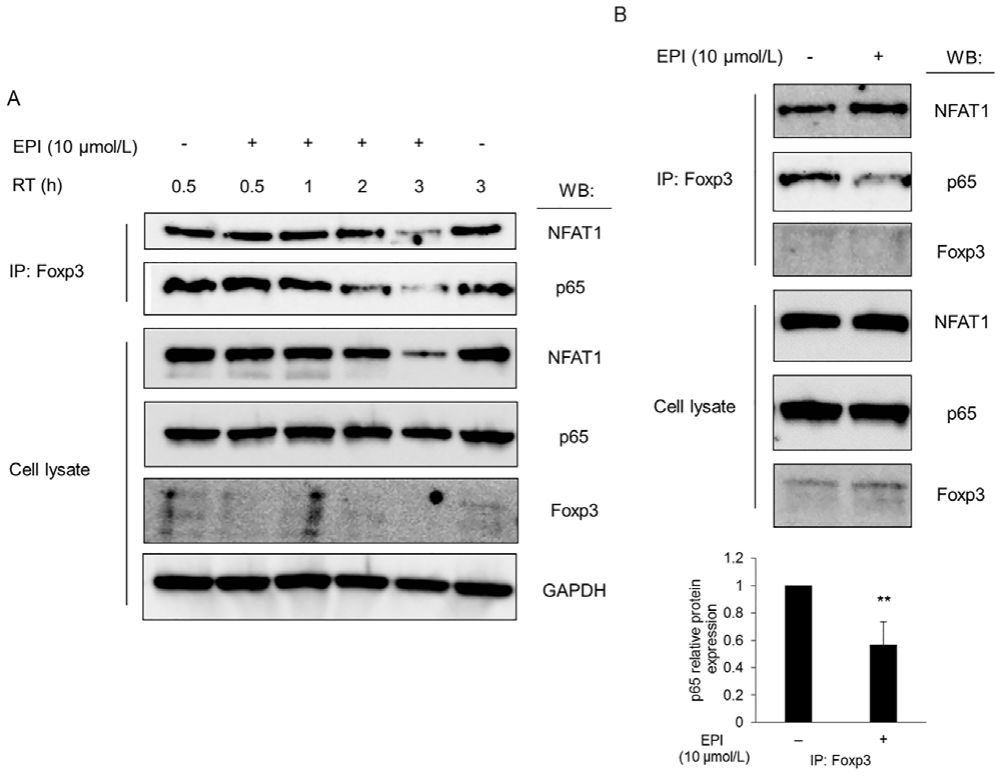
Epirubicin (EPI) blocks the physical interaction between Foxp3 and the p65 subunit of NF-κB. Proteins were run on SDS-PAGE and immunoblotted with anti-NFAT1, anti-p65 or anti-Foxp3 antibody to detect the total cell lysates or immunoprecipitates (IP). (A) Karpas-299 cells were incubated for the indicated times (RT) with epirubicin (10 μmol/L). (B) *In vitro* immunoprecipitation (cell-free assay) was performed by incubating the Karpas-299 total cell lysates with epirubicin (10 μmol/L) and anti-Foxp3 antibody at 4°C for 4 h. p65 protein expression levels were determined by densitometry as values normalized relative to the control (the lysates incubated with DMSO) and expressed as the mean ± SD from three independent experiments. Asterisks represent statistically significant differences as determined by Student’s *t* test (**, *p*< 0.01).

A previous study showed that Foxp3 physically associates with NFAT and the p65 subunit of NF-κB and represses their ability to transactivate [17]. We therefore tested the possibility that epirubicin could inhibit the Foxp3-NFAT or Foxp3-p65 interaction. Karpas-299, a human T cell lymphoma cell line that expresses Foxp3 and shows characteristics typical of Tregs, was used [20]. Immunoblotting of total cell lysates confirmed that treatment of Karpas-299 cells with epirubicin at 10 μmol/L for 0.5–3h did not affect the expression levels of Foxp3 or p65. Interestingly, immunoprecipitation using anti-Foxp3 antibody revealed that epirubicin treatment decreased the amount of p65 protein associated with Foxp3 (Fig 3A), indicating that epirubicin inhibited the physical interaction between Foxp3 and p65 without modulating their expression. Similar analysis was also performed on another Foxp3-binding transcriptional factor, NFAT. Higher concentrations of epirubicin reduced the amount of NFAT co-precipitated with Foxp3; however, this occurred in parallel with a decrease in the total amount of NFAT (Fig 3A), making it difficult to determine the effect of epirubicin specifically on the interaction between Foxp3 and NFAT.

To further explore the possibility of inhibition of binding between Foxp3 and p65 by epirubicin, we next conducted a cell-free experiment; incubation of a Karpas-299 total cell lysates containing Foxp3 and p65 with epirubicin at 10 μmol/L followed by immunoprecipitation with anti-Foxp3 antibody (Fig 3B). Again, epirubicin reduced the amount of p65 co-precipitated with Foxp3, providing further evidence that epirubicin directly abrogated the interaction between these transcription factors. It should also be noted that epirubicin did not affect the co-precipitation of NFAT with Foxp3 in this cell-free assay (Fig 3B), indicating that epirubicin was likely to have decreased the interaction between NFAT and Foxp3 by reducing NFAT expression rather than via direct inhibition of their interaction. Together, these results clearly suggest that epirubicin specifically blocked the interaction between Foxp3 and p65, and could thereby restore the transactivation activity of p65.

### Epirubicin inhibits the immunosuppressive activity of murine Tregs *in vitro*

**Fig 4:**
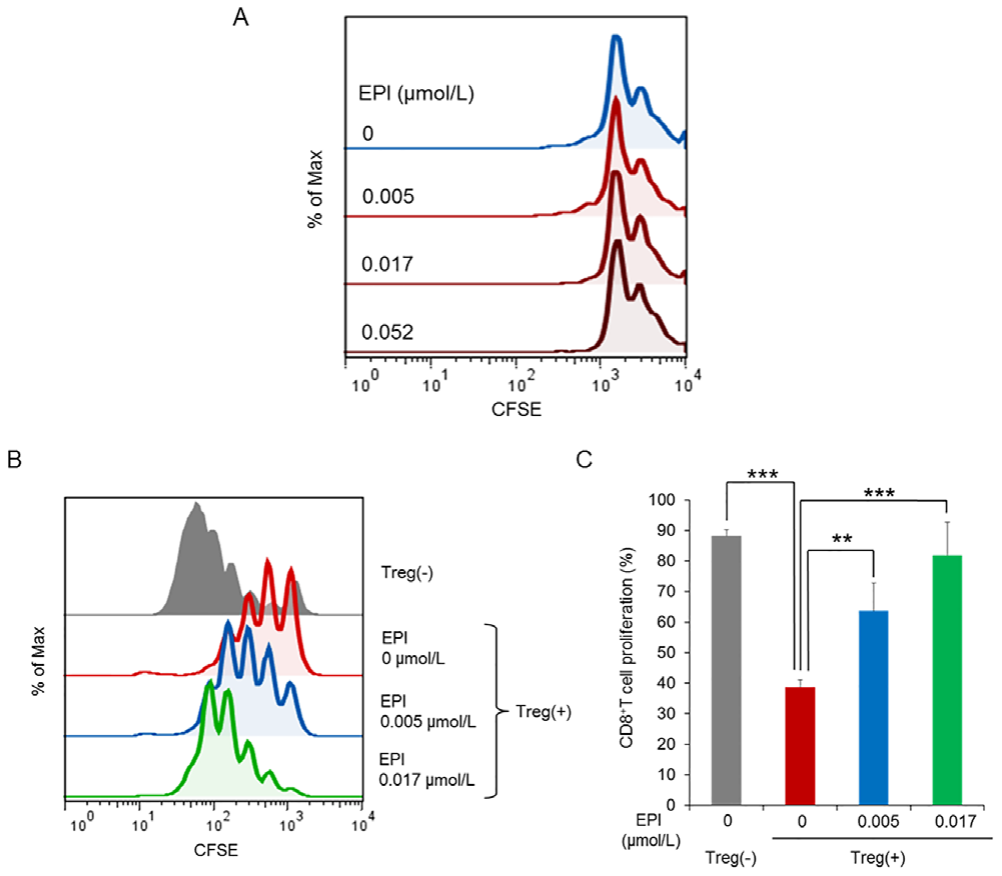
Epirubicin (EPI) inhibits the immunosuppressive activity of murine Tregs *in vitro*. (A) Tregs were isolated from BALB/c mouse spleens and were cultured for a total of 48 h with plate-bound anti-CD3, soluble anti-CD28 and IL-2 with epirubicin at the indicated concentrations. Effects of epirubicin on Treg proliferation was determined by the CFSE method. CFSE proliferation profile shows representative result of two independent experiments. (B) Tregs from spleens of BALB/c mice were exposed to increasing but non-toxic concentrations of epirubicin for 48 h, recovered and washed three times in complete medium to eliminate residual epirubicin. The effect of treated or untreated Tregs on the proliferation of stimulated responder CD8^+^ T cells was determined by the CFSE method. CFSE proliferation profile depicts a representative data. (C) CD8^+^ T cell proliferation was analyzed using CFSE proliferation profile (Fig 4B). Each column represents the mean ± SD (n = 3). Asterisks represent statistically significant differences as determined by Dunnett’s test (**, *p*< 0.01; ***, *p*< 0.001).

We next sought to investigate the effect of epirubicin on the function of Foxp3 under more physiological conditions by using primary Tregs instead of cell lines. Specifically, we tested the influence of epirubicin on Treg proliferation *in vitro*. CD45^+^CD25^+^ Tregs isolated from mouse spleens were stained with a fluorescent dye CFSE, and stimulated with anti-CD3 Ab, anti-CD28 Ab and IL-2 in the presence of epirubicin at different concentrations for 48h. Treg proliferation was determined by measuring CFSE dilution using a flow cytometer. We found that Treg proliferation was not affected by epirubicin treatment (Fig 4A). To further clarify the effects of epirubicin on murine Tregs, suppression of CD8^+^ T cell proliferation by epirubicin-treated or untreated Tregs was examined. Tregs isolated from mouse spleens were first exposed to epirubicin under stimulation with anti-CD3 Ab, anti-CD28Ab and IL-2 for 48 h. After washing out epirubicin, CD8^+^T cells stained with CFSE were then added to Tregs and stimulated with anti-CD3 Ab and anti-CD28 Ab. After 72 h, CD8^+^ T cell proliferation was determined by measuring CFSE dilution (Fig 4B). The analytical data depicted in Fig 4C indicates that epirubicin inhibited significantly the capacity of Tregs to suppress CD8^+^ T cell proliferation.

### Epirubicin might modulate immunosuppressive function of Tregs *in vivo*

**Fig 5:**
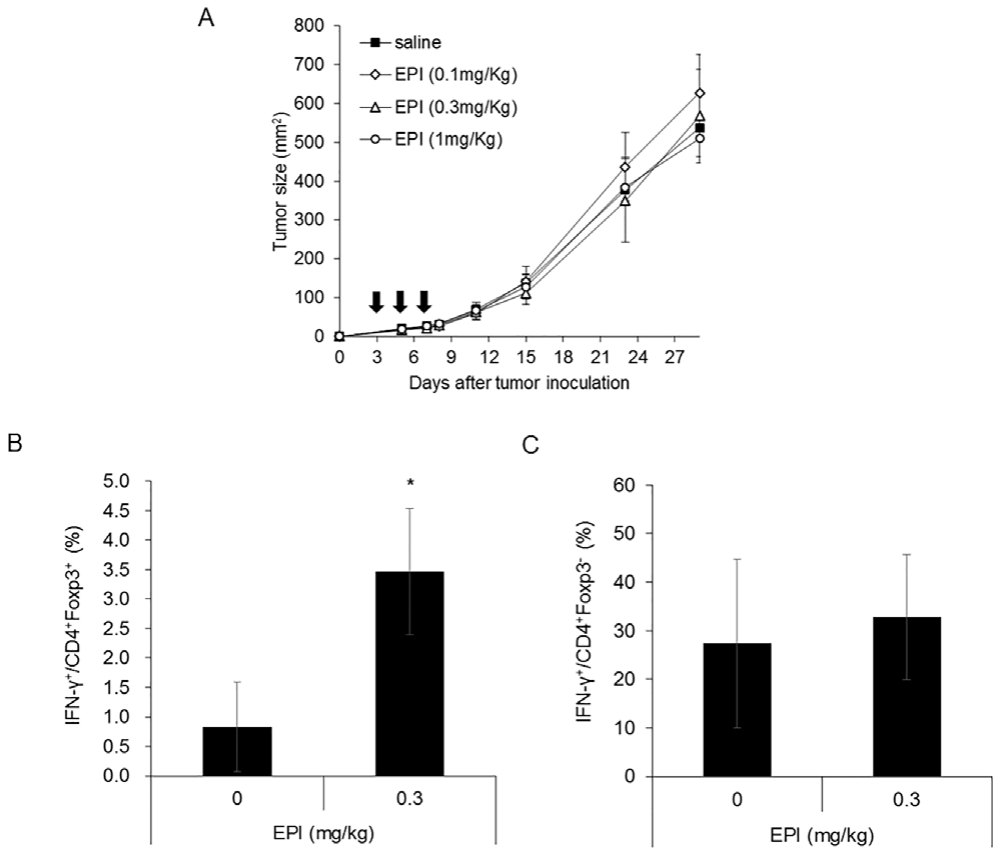
Epirubicin (EPI) might modulate immunosuppressive function of Tregs *in vivo*. (A) CMS5a tumor cells (1 x10^6^) were injected (s.c.) in the right inguinal region of BALB/c mice (8 mice per group). Mice were treated with epirubicin (0.1, 0.3 or 1 mg/kg i.v. dosing on days 3, 5 and 7 as indicated by arrows) or saline, and tumor volume was measured at appropriate intervals until day 29. The data are shown as the mean ± SD (n = 4). (B) The percentage of IFN-γ-positive cells within the CD4^+^FoxP3^+^ cell population. (C) The percentage of IFN-γ-positive cells within the CD4^+^FoxP3^-^ cell population. On day 8, the mice treated with epirubicin or saline were euthanized and tumors were removed. Tumor-infiltrating lymphocytes dissociated from the pooled tumors were stained with anti-CD4, anti-CD8, anti-Foxp3 and anti-IFN-γ antibodies, and then analyzed by flow cytometry. Each column represents the mean ± SD (n = 3). Asterisks represent statistically significant differences as determined by Student’s *t* test (*, *p*< 0.05).

We further evaluated the influence of epirubicin on immune cells using tumor-bearing mice. BALB/c mice subcutaneously inoculated with mouse fibrosarcoma CMS5a cells, were given intravenous injections of epirubicin for 7 days. To accurately identify the immunological effect of epirubicin, this evaluation was performed at low doses, from 0.1 to 1 mg/kg, having no impact on tumor growth compared with untreated animals (Fig 5A). Tumors were removed 1 day after the last injection of epirubicin. Tumor-infiltrating lymphocytes dissociated from the tumors were stained with anti-CD4, anti-CD8, anti-Foxp3 and anti-IFN-γ antibodies, and then analyzed by flow cytometry. The percentage of IFN-γ-positive cells within the CD4^+^Foxp3^+^ and the CD4^+^Foxp3^-^ populations was determined (Fig 5B and 5C). Interestingly, the percentage of IFN-γ-positive cells within the CD4^+^Foxp3^+^ cell population was significantly increased in the mice treated with epirubicin. The percentage of IFN-γ-positive cells within the CD4^+^Foxp3^-^ cell population was not changed. These results demonstrate that epirubicin could modulate the function of immune cells by affecting Foxp3 and promotes the production of IFN-γ in the tumor microenvironment without exerting cytotoxicity.

## Discussion

The original purpose of this work was to identify novel small molecular Foxp3 inhibitors that modulate the function of Tregs. As a screening system, we adopted a cell-based screen using stable HEK293/NF-κB-RE/Foxp3 cells, and probed for reversal of the suppression of the NF-κB reporter signal by Foxp3. It has been shown that peptide P60 overcomes the inhibitory effect of Foxp3 in this assay system, and indeed impairs Treg activity *in vitro* and *in vivo* [13]. We therefore thought that this screen system was suitable to find novel small molecular Foxp3 inhibitors. There have been no previous reports on the application of this approach for screening purposes. In screens that seek to identify downregulated responses, off-target cytotoxicity of compounds can be problematic because it can reduce reporter gene expression and can give false positives, making it difficult to appropriately interpret obtained result. In our Foxp3 inhibitor screening system, inhibitory activity of compounds is detected as an upregulated response, allowing us to screen the library with high efficiency. In our attempt to identify novel Foxp3 inhibitors, epirubicin was the only compound that restored NF-κB activity in the face of suppression by Foxp3 and matched with our concept of Foxp3 inhibitors. Although anthracyclines are reported to stimulate the activity of NF-κB through IκB-α degradation [21, 22], epirubicin did not enhance the signal in the absence of Foxp3 in our NF-κB reporter assay. Considering that epirubicin successfully inhibited the function of murine Tregs, our luciferase-based screen for Foxp3 inhibitors could represent a useful surrogate assay for identifying compounds that inhibit Treg suppressor function. Together our findings suggest that while the identification of novel small molecular Foxp3 inhibitors is challenging, our cell-based screening approach is valid and screening larger numbers of compounds from various sources is likely to be worthwhile.

Following the identification of epirubicin as a Foxp3 inhibitor, we first wondered whether epirubicin had the ability to block protein-protein interactions (PPI). In support of this possibility, a recent report indicated that doxorubicin might disrupt the PPI between Dst1 (a transcription elongation factor for RNA polymerase II) and Rpb9 (a subunit of RNA polymerase II) [23]. Our results showed that epirubicin inhibited the physical interaction between Foxp3 and the p65 subunit of NF-κB. However, from our work it is not clear which is their interaction site cytoplasmic or nuclear. A previous report showed that Foxp3 inhibits nuclear translocation of NF-κB by increasing the stability of IκB-α which interacts with p65 [24]. Besides their direct action at NF-κB binding sites in nuclear, it is thus likely that the repression of PPI between the two molecules by epirubicin indirectly decreases the stability of IκB-α, resulting in acceleration of NF-κB nuclear translocation. Meanwhile, it has been revealed that Foxp3 can form a dynamic supermolecular complex consistent of a variety of transcription factors and enzymatic proteins [25–27]. This indicates the possibility that epirubicin interacts with many other proteins in addition to p65 and NFAT, which we focused on. We note that disruption of PPI between Foxp3 and p65 by epirubicin might, partially at least, contribute to the modulation of Treg function. Despite the vital role of Foxp3 in the function of Tregs, its mechanism of action currently remains unclear [25]. To determine the overall mechanism of action by which epirubicin modulates Treg function, further structural and biochemical investigation is certainly warranted.

Anthracyclines are chemotherapeutic agents that are effective against a broad spectrum of solid tumors and leukemias [28]. Despite their longstanding and widespread clinical use, the exact mode of action of anthracyclines is not fully understood. The common understanding is that anthracyclines work primarily by intercalating with DNA strands and then inhibiting topoisomerase II [29], however the data collected to date implies the contribution of other mechanisms as well [30]. Also, recent increasing evidence suggests that conventional chemotherapeutics affect the immune system in various manner [31–34]. In light of these findings, it is noteworthy that it has been reported that epirubicin may improve immune function by inhibiting the secretion of soluble CD25 by Tregs isolated from diffuse large B-cell lymphoma patients [35]. Our *in vivo* data showed that IFN-γ production from immune cells was promoted by epirubicin. However, the impact on other cytokines such as IL-10 and IL-2 and other immune cells such as CD8^+^ T cells remained unknown. Further studies are needed in order to uncover *in vivo* effect of epirubicin for Tregs.

In summary, we identified the novel action of epirubicin as a Foxp3 inhibitor using a newly established luciferase-based cellular screen. Low dose epirubicin inhibited Treg activity and modulated the function of immune cells *in vivo*. Our work also indicated that our luciferase-based screen might be useful in accelerating the discovery of Foxp3 inhibitors.

## Supporting Information

S1 Fig Development of a new cell-based screen that detects the reversal of NF-κB reporter signal suppression by Foxp3. (A) HEK293/NF-κB-RE/Foxp3 cells were stimulated with 10 ng/mL TNF-α for 5h. Foxp3 mRNA levels were measured by quantitative RT-PCR. Foxp3/GAPDH mRNA ratio (%) was calculated as a percentage of control cells (HEK 293/pcDNA3.1-Foxp3 cells). (B) HEK293/NF-κB-RE/Foxp3 or HEK293/NF-κB-RE cells were incubated for 24 h with peptide P60 (10, 50 or 100 μmol/L) and then stimulated with 0.3 ng/mL TNF-α for 2.5 h, followed by detection of NF-κB-dependent luciferase activity. Each column represents the mean (n = 2).
(TIF)

S2 Fig Chemical structure of anthracycline derivatives. (A) epirubicin. (B) doxorubicin, pirarubicin, daunorubicin and idarubicin.
(TIF)
