# Supplementary figures and images for: Epirubicin, Identified Using a Novel Luciferase Reporter Assay for Foxp3 Inhibitors, Inhibits Regulatory T Cell Activity
Source: PLoS One. 2016 Jun 10;11(6):e0156643. doi: 10.1371/journal.pone.0156643 (PMC4902191; doi:10.1371/journal.pone.0156643)

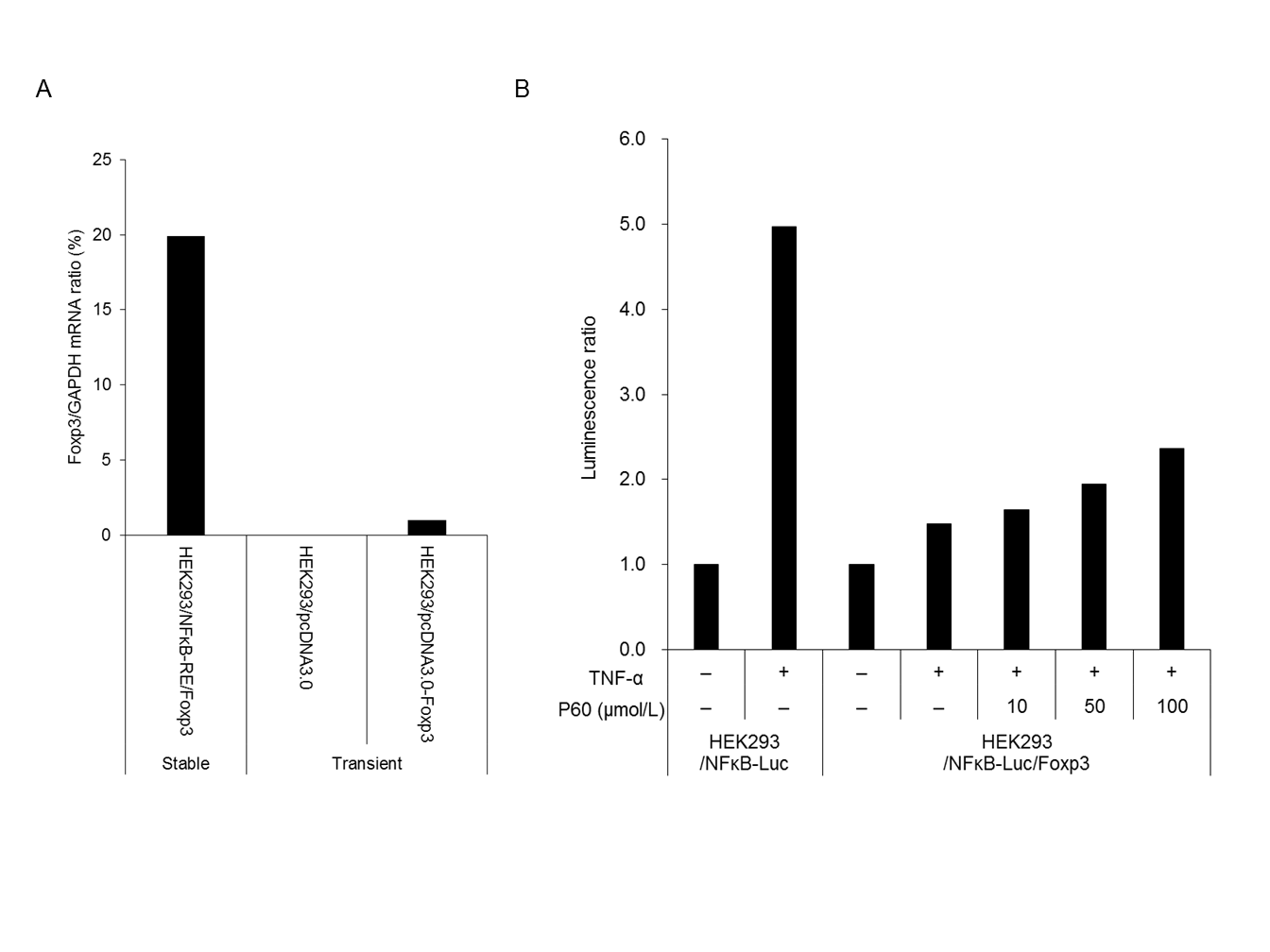

Supplement: S1 Fig — (A) HEK293/NF-κB-RE/Foxp3 cells were stimulated with 10 ng/mL TNF-α for 5h. Foxp3 mRNA levels were measured by quantitative RT-PCR. Foxp3/GAPDH mRNA ratio (%) was calculated as a percentage of control cells (HEK 293/pcDNA3.1-Foxp3 cells). (B) HEK293/NF-κB-RE/Foxp3 or HEK293/NF-κB-RE cells were incubated for 24 h with peptide P60 (10, 50 or 100 μmol/L) and then stimulated with 0.3 ng/mL TNF-α for 2.5 h, followed by detection of NF-κB-dependent luciferase activity. Each column represents the mean (n = 2). (TIF) [file pone.0156643.s001.tif]

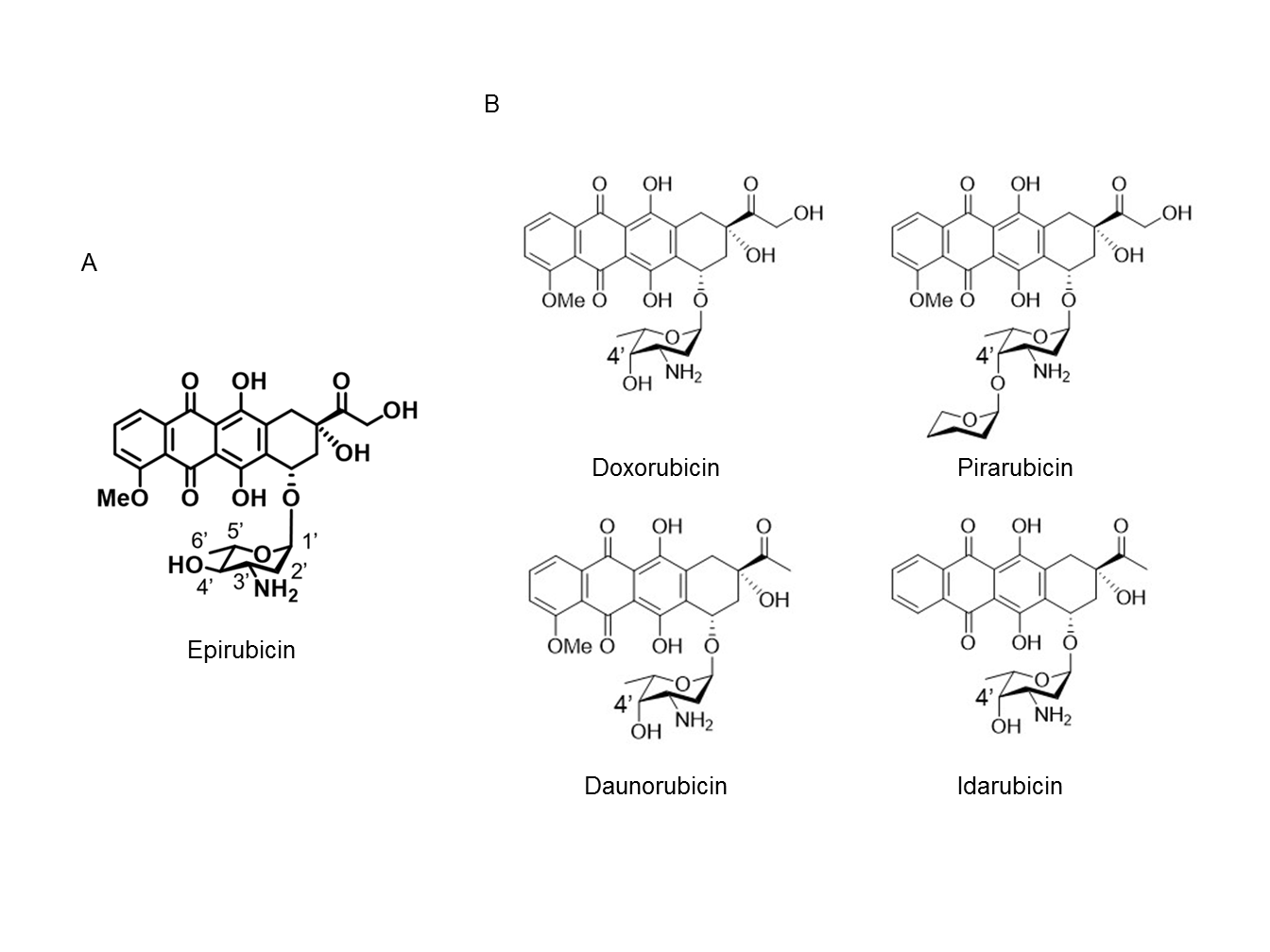

Supplement: S2 Fig — (A) epirubicin. (B) doxorubicin, pirarubicin, daunorubicin and idarubicin. (TIF) [file pone.0156643.s002.tif]
